# Biallelic variants in *SNUPN* cause a limb girdle muscular dystrophy with myofibrillar-like features

**DOI:** 10.1093/brain/awae046

**Published:** 2024-02-15

**Authors:** Pablo Iruzubieta, Alberto Damborenea, Mihaela Ioghen, Simon Bajew, Roberto Fernandez-Torrón, Ana Töpf, Álvaro Herrero-Reiriz, Diana Epure, Katharina Vill, Aurelio Hernández-Laín, María Manterola, Mikel Azkargorta, Oihane Pikatza-Menoio, Laura Pérez-Fernandez, Mikel García-Puga, Gisela Gaina, Alexandra Bastian, Ioana Streata, Maggie C Walter, Wolfgang Müller-Felber, Simone Thiele, Saioa Moragón, Nerea Bastida-Lertxundi, Aitziber López-Cortajarena, Felix Elortza, Gorka Gereñu, Sonia Alonso-Martin, Volker Straub, David de Sancho, Raluca Teleanu, Adolfo López de Munain, Lorea Blázquez

**Affiliations:** Department of Neurosciences, Biogipuzkoa Health Research Institute, 20014 San Sebastián, Spain; Department of Neurology, Donostia University Hospital, Osakidetza Basque Health Service, 20014 San Sebastián, Spain; CIBERNED, ISCIII (CIBER, Carlos III Institute, Spanish Ministry of Sciences and Innovation), 28031, Madrid, Spain; Department of Neurosciences, Biogipuzkoa Health Research Institute, 20014 San Sebastián, Spain; Clinical Neurosciences Department, Faculty of Medicine, Carol Davila University of Medicine and Pharmacy, Paediatric Neurology, 020021 Bucharest, Romania; Department of Neurosciences, Biogipuzkoa Health Research Institute, 20014 San Sebastián, Spain; Department of Neurosciences, Biogipuzkoa Health Research Institute, 20014 San Sebastián, Spain; Department of Neurology, Donostia University Hospital, Osakidetza Basque Health Service, 20014 San Sebastián, Spain; John Walton Muscular Dystrophy Research Centre, Newcastle University and Newcastle Hospitals NHS Foundation Trust, NE4 5NR Newcastle Upon Tyne, UK; Department of Neurosciences, Biogipuzkoa Health Research Institute, 20014 San Sebastián, Spain; Department of Paediatric Neurology, Doctor Victor Gomoiu Children’s Hospital, 022102 Bucharest, Romania; Department of Pediatric Neurology and Developmental Medicine and LMU Center for Children with Medical Complexity, Dr. von Hauner Children’s Hospital, LMU University Hospital, Ludwig-Maximilians-University Munich, 80539 Munich, Germany; Institute of Human Genetics, School of Medicine, Technical University of Munich, 81675 Munich, Germany; Neuropathology Unit, Department of Pathology, 12 de Octubre University Hospital, 28041 Madrid, Spain; Department of Neuro-oncology, Instituto de Investigación Sanitaria imas12, Hospital Universitario 12 de Octubre, 28041 Madrid, Spain; Universidad Complutense de Madrid, Facultad de Medicina, 28040 Madrid, Spain; Department of Neurosciences, Biogipuzkoa Health Research Institute, 20014 San Sebastián, Spain; Proteomics Platform, CIC bioGUNE, Basque Research and Technology Alliance (BRTA), 48160 Derio, Spain; Centre for the Study of Liver and Gastrointestinal Diseases (CIBERehd), Instituto de Salud Carlos III (ISCIII), 28029 Madrid, Spain; Department of Neurosciences, Biogipuzkoa Health Research Institute, 20014 San Sebastián, Spain; CIBERNED, ISCIII (CIBER, Carlos III Institute, Spanish Ministry of Sciences and Innovation), 28031, Madrid, Spain; Department of Neurosciences, Biogipuzkoa Health Research Institute, 20014 San Sebastián, Spain; Center for Cooperative Research in Biomaterials (CIC biomaGUNE), Basque Research and Technology Alliance (BRTA), 20014 San Sebastián, Spain; Department of Neurosciences, Biogipuzkoa Health Research Institute, 20014 San Sebastián, Spain; CIBERNED, ISCIII (CIBER, Carlos III Institute, Spanish Ministry of Sciences and Innovation), 28031, Madrid, Spain; Department of Cell Biology, Neurosciences and Experimental Myology, Victor Babes National Institute of Pathology, 050096 Bucharest, Romania; Department of Pathology, Colentina Clinical Hospital, 020125 Bucharest, Romania; Human Genomics Laboratory, Regional Centre of Medical Genetics, Craiova University of Medicine and Pharmacy, 200349 Dolj, Romania; Friedrich Baur Institute at the Department of Neurology, LMU University Hospital, Ludwig-Maximilians-University Munich, 80539 Munich, Germany; Institute of Human Genetics, School of Medicine, Technical University of Munich, 81675 Munich, Germany; Friedrich Baur Institute at the Department of Neurology, LMU University Hospital, Ludwig-Maximilians-University Munich, 80539 Munich, Germany; Department of Neurosciences, Biogipuzkoa Health Research Institute, 20014 San Sebastián, Spain; Department of Clinical Genetics, Donostia University Hospital, Osakidetza Basque Health Service, 20014 San Sebastián, Spain; Center for Cooperative Research in Biomaterials (CIC biomaGUNE), Basque Research and Technology Alliance (BRTA), 20014 San Sebastián, Spain; Ikerbasque, Basque Foundation for Science, 48009 Bilbao, Spain; Proteomics Platform, CIC bioGUNE, Basque Research and Technology Alliance (BRTA), 48160 Derio, Spain; Centre for the Study of Liver and Gastrointestinal Diseases (CIBERehd), Instituto de Salud Carlos III (ISCIII), 28029 Madrid, Spain; Department of Neurosciences, Biogipuzkoa Health Research Institute, 20014 San Sebastián, Spain; CIBERNED, ISCIII (CIBER, Carlos III Institute, Spanish Ministry of Sciences and Innovation), 28031, Madrid, Spain; Ikerbasque, Basque Foundation for Science, 48009 Bilbao, Spain; Department of Neurosciences, Biogipuzkoa Health Research Institute, 20014 San Sebastián, Spain; CIBERNED, ISCIII (CIBER, Carlos III Institute, Spanish Ministry of Sciences and Innovation), 28031, Madrid, Spain; John Walton Muscular Dystrophy Research Centre, Newcastle University and Newcastle Hospitals NHS Foundation Trust, NE4 5NR Newcastle Upon Tyne, UK; Donostia International Physics Center, 20018 San Sebastián, Spain; Faculty of Chemistry, University of the Basque Country, 20018 San Sebastián, Spain; Clinical Neurosciences Department, Faculty of Medicine, Carol Davila University of Medicine and Pharmacy, Paediatric Neurology, 020021 Bucharest, Romania; Department of Neurosciences, Biogipuzkoa Health Research Institute, 20014 San Sebastián, Spain; Department of Neurology, Donostia University Hospital, Osakidetza Basque Health Service, 20014 San Sebastián, Spain; CIBERNED, ISCIII (CIBER, Carlos III Institute, Spanish Ministry of Sciences and Innovation), 28031, Madrid, Spain; Faculty of Medicine, University of the Basque Country, 20014 San Sebastián, Spain; Faculty of Medicine, University of Deusto, 48007 Bilbao, Spain; Department of Neurosciences, Biogipuzkoa Health Research Institute, 20014 San Sebastián, Spain; CIBERNED, ISCIII (CIBER, Carlos III Institute, Spanish Ministry of Sciences and Innovation), 28031, Madrid, Spain; Ikerbasque, Basque Foundation for Science, 48009 Bilbao, Spain

**Keywords:** snurportin-1, small-nuclear ribonucleoproteins (snRNPs), splicing, myopathy

## Abstract

Alterations in RNA-splicing are a molecular hallmark of several neurological diseases, including muscular dystrophies, where mutations in genes involved in RNA metabolism or characterized by alterations in RNA splicing have been described. Here, we present five patients from two unrelated families with a limb-girdle muscular dystrophy (LGMD) phenotype carrying a biallelic variant in *SNUPN* gene.

Snurportin-1, the protein encoded by *SNUPN*, plays an important role in the nuclear transport of small nuclear ribonucleoproteins (snRNPs), essential components of the spliceosome. We combine deep phenotyping, including clinical features, histopathology and muscle MRI, with functional studies in patient-derived cells and muscle biopsies to demonstrate that variants in *SNUPN* are the cause of a new type of LGMD according to current definition. Moreover, an *in vivo* model in *Drosophila melanogaster* further supports the relevance of Snurportin-1 in muscle.

*SNUPN* patients show a similar phenotype characterized by proximal weakness starting in childhood, restrictive respiratory dysfunction and prominent contractures, although inter-individual variability in terms of severity even in individuals from the same family was found. Muscle biopsy showed myofibrillar-like features consisting of myotilin deposits and Z-disc disorganization. MRI showed predominant impairment of paravertebral, vasti, sartorius, gracilis, peroneal and medial gastrocnemius muscles. Conservation and structural analyses of Snurportin-1 p.Ile309Ser variant suggest an effect in nuclear-cytosol snRNP trafficking. In patient-derived fibroblasts and muscle, cytoplasmic accumulation of snRNP components is observed, while total expression of Snurportin-1 and snRNPs remains unchanged, which demonstrates a functional impact of *SNUPN* variant in snRNP metabolism. Furthermore, RNA-splicing analysis in patients’ muscle showed widespread splicing deregulation, in particular in genes relevant for muscle development and splicing factors that participate in the early steps of spliceosome assembly.

In conclusion, we report that *SNUPN* variants are a new cause of limb girdle muscular dystrophy with specific clinical, histopathological and imaging features, supporting *SNUPN* as a new gene to be included in genetic testing of myopathies. These results further support the relevance of splicing-related proteins in muscle disorders.

## Teaser

Iruzubieta *et al.* identify a new type of limb girdle muscular dystrophy, characterised by progressive muscle weakness and atrophy. Using clinical and molecular data, they show that the disorder is caused by mutations in Snurportin-1, a protein involved in the nuclear transport of essential components of the spliceosome.

## Introduction

Muscular dystrophies are a complex and heterogeneous group of neuromuscular disorders characterized by progressive muscle weakness and atrophy caused by loss of muscle fibres.^1^ Limb girdle muscular dystrophies (LGMD) are a group of muscular dystrophies with predominantly proximal muscle weakness at presentation.^2^ Genetic variants are the main cause of muscular dystrophies, mainly affecting proteins essential for several muscular functions. Interestingly, splicing related genes have also been shown to be the cause of specific types of muscular dystrophies.^3,4^ Despite the increasing number of genes related to these disorders and the technical advances, around half of patients with LGMD remain without a genetic diagnosis.^5^

Precursor messenger RNA (pre-mRNA) splicing is an essential step in the generation of mature mRNA transcripts. It serves as a mechanism that regulates gene expression by intron removal and exon binding. Splicing is performed by a large and highly dynamic ribonucleoprotein (RNP) complex, the spliceosome, which contains both RNAs and proteins. The U1, U2, U4/U6 and U5 small nuclear RNPs (snRNPs) are the main building blocks of the major spliceosome, which is responsible for removing the vast majority of pre-mRNA introns. They are composed of U-rich small nuclear RNAs (U-snRNAs) bound to specific RNA-binding proteins (RBPs)^6-8.^

The biogenesis of snRNPs from the major spliceosome involves transcription of U-snRNA genes in the nucleus by RNA polymerase II and co-transcriptional acquisition of a 7-methylguanosine (m^7^G) cap structure at their 5´ end. The newly transcribed snRNAs are then actively exported to the cytoplasm assisted by the cap-binding complex (CBC). In the cytoplasm, they undergo three additional processing steps. First, Sm proteins are arranged around the Sm binding site of the U-snRNA by the SMN complex, constituting the Sm core. Subsequently, the m^7^G monomethyl cap is hypermethylated to a 2,2,7-trimethylguanosine (TMG) cap by TGS1 enzyme and finally U-snRNAs undergo 3´ end maturation. These maturation steps trigger U-snRNP import back into the nucleus, in a process mediated by Importin-β in TMG cap-dependent and independent pathways.^9,10^ Snurportin-1 is an RBP that functions as an snRNP-specific nuclear import adapter in a TMG cap-dependent manner. Snurportin-1 contains three functional domains, including an N-terminal Importin-β binding (IBB) domain, a centrally located TMG cap binding domain, necessary for snRNP import into the nucleus,^11-14^ and a less precisely defined region responsible for binding to Exportin-1, which mediates its recycling back into the cytoplasm^14-17^.

Here, we present five affected individuals from two unrelated families carrying a biallelic variant in *SNUPN*, which encodes Snurportin-1, showing a LGMD phenotype characterized by early-onset proximal weakness, restrictive respiratory dysfunction and frequent contractures. Our findings indicate that deleterious variants in *SNUPN* gene are the cause of a novel form of muscular dystrophy characterized by alterations in snRNP biogenesis and widespread splicing deregulation.

## Materials and methods

### Clinical and genetic studies

Samples and data from all subjects included in the study were collected after obtention of their informed consent or that from their legal guardians. Research was performed according to international guidelines for studies with human subjects and materials and ethical approval was granted by the National Research Ethics Service (NRES) Committee North East–Newcastle and North Tyneside 1 (reference 19/NE/0028). Affected individuals were investigated according to routine clinical standards for the diagnosis of neuromuscular disease.

Muscle biopsies from patients were obtained for diagnostic purposes and processed following standard histological protocols, as previously described.^18^ Muscle biopsies from controls (*n* = 3) were obtained from healthy individuals undergoing accident-related surgeries in the Traumatology Department of Donostia University Hospital (Spain). Skin biopsies were obtained from two patients (Patients F1.II.1 and F1.II.2) and two healthy controls. Fibroblasts were isolated according to standard clinical procedures. More information about the samples is provided in Supplementary Table 1. Muscle MRI studies were performed in a 1.5 T scan. FSE T_1_-weighted and T_2_-weighted images were obtained. T_1_-weighted images were selected to evaluate muscle fat replacement.

Genomic DNA and RNA from blood was isolated using QIAamp® DNA and RNeasy Mini Kits (Qiagen). In both families, genomic DNA was subjected for whole exome sequencing (WES). WES was performed by Centogene (https://www.centogene.com) in Family 1 or within the MYOSEQ project^5^ in Family 2. Segregation studies were performed by standard PCR procedures and Sanger sequencing using the list of primers described in Supplementary Table 2. Primers used are described in Supplementary Table 2. Variant frequency was assessed in genomAD v4 and *in silico* deleteriousness prediction was performed using Polyphen (http://genetics.bwh.harvard.edu/pph2/), SIFT (https://sift.bii.a-star.edu.sg/), Combined Annotation Dependent Depletion (CADD) (https://cadd.gs.washington.edu/) and AlphaMissense.^19^

### Protein conservation and structural analysis

The Protein Data Bank (PDB) was queried for experimental structures of Snurportin-1 (Uniprot id: O95149), which included nuclear import (PDB ids: 2p8q, 2q5d, 2qna and 3lww) and export complexes (PDB ids: 3gb8, 3gjx, 3nby, 3nbz, 3nc0, 5dis) and an experimental structure of free Snurportin-1 bound to a dinucleotide substrate (PDB id: 1xk5). In the import complex structures, the only resolved part was the N-terminal IBB domain (residues 11–73 according to InterPro),^20^ while the export complexes also contained coordinates for the central m3G-cap-binding domain (residues 97–280). The only structural model containing atomic coordinates for all the protein residues is that predicted by the Alphafold algorithm, which is available at the Alphafold Protein Structure Database.^21,22^ Prediction of intrinsically disordered regions was performed using multiple prediction servers.^23-25^

Conservation of the mutated residue was studied by multiple sequence alignment of the amino acid sequence of Snurportin-1 in representative species of the subphylum Vertebrata and *Drosophila melanogaster*, which was included as an outgroup. Alignment was performed with ClustalWS (default settings) using the Jalview software.^26^

### Cell culture, RNA extraction and reverse transcription quantitative PCR

Fibroblasts were cultured in Dulbecco’s modified Eagle medium (DMEM) supplemented with 10% inactivated foetal bovine serum and 1% penicillin-streptomycin (Gibco/Life Technologies). For reverse transcription quantitative PCR (RT-qPCR), RNA was isolated with the Maxwell® RSC simplyRNA Cells Kit (Promega) and reverse transcribed with the High-Capacity cDNA Reverse Transcription Kit (Applied Biosystems). The qPCRs were performed using SYBR™ Green PCR Master Mix (Applied Biosystems). The relative changes in gene expression were analysed with the 2-ΔΔCt method, using *TBP* expression as housekeeping and normalized to the Control 2 sample. Primers used for these studies are shown in Supplementary Table 2.

### Western-blot and immunofluorescence

For western blot, 1 000 000 cells were collected, resuspended in ice-cold RIPA buffer (Sigma Aldrich), supplemented with complete™ Protease Inhibitor Cocktail (Roche) and further lysed by freeze-thawing cycles. Nuclear/cytoplasmic fractionation was performed using the NE-PER® Nuclear and Cytoplasmic Extraction Reagent Kit (Thermo Scientific) according to the manufacturer’s instructions. Protein concentration was quantified using the DC Protein Assay (Bio-Rad Laboratories). Total, cytoplasmic or nuclear fractions were separated by protein electrophoresis in NuPAGE™ 4–12%, Bis-Tris Gels (InvitroGen) and transferred to nitrocellulose membranes. Proteins of interest were detected using the antibodies provided in Supplementary Table 3. Blots were imaged using the iBright FL1000 imaging system (Thermo Scientific) and signal intensities were quantified using the iBright Analysis Software (Thermo Scientific). Band intensities were normalized to α-tubulin in each blot and protein expression was represented relative to Control 1 sample.

For immunofluorescence, frozen muscle sections were fixed in 4% paraformaldehyde (Thermo Scientific) and permeabilized in PBS with 0.5% Triton-X-100. Samples were then blocked using a blocking solution [5% bovine serum albumin (BSA), 10% goat serum and 0.025 Tween 20] for 2 h and, afterwards, incubated in primary antibodies (Supplementary Table 3) overnight at 4°C. The day after, three washes in PBS were performed and fluorescent secondary antibodies added (Supplementary Table 3). Samples were mounted using Fluoromount mounting medium (Thermo Scientific).

### RNA sequencing

Total RNA for RNA sequencing was isolated from ∼30 mg of total muscle using the QIAzol® lysis reagent (QIAGEN) following standard procedures. Muscle tissue was first homogenized on the OMNI Bead Ruptor 12 (OMNI International) bead mill homogenizer (six cycles, on for 30 s at 6 m/s and six cycles off for 5 min on ice). DNase I treatment and RNA clean-up was performed with RNeasy Mini Kit (QIAGEN) according to the manufacturer’s instructions. Stranded full-length total RNA library preparation was performed at BGI Genomics (China) with the Ribo-Zero Plus rRNA depletion kit (Illumina). Single-end RNA sequencing was performed on Illumina NextSeq2000 platform with a sequencing read length of 100 nt.

### Proteomics

Protein was extracted by incubating the extracts in a buffer containing 7 M urea, 2 M thiourea and 4% CHAPS. Samples were incubated in this buffer for 30 min at room temperature under agitation and digested following the FASP protocol previously described.^27^ Trypsin was added in 50 mM ammonium bicarbonate to a trypsin:protein ratio of 1:10, and the mixture was incubated overnight at 37°C. Peptides were dried out in an RVC2 25 speedvac concentrator (Christ) and resuspended in 0.1% FA. Peptides were desalted and resuspended in 0.1% FA using C18 stage tips (Millipore) prior to acquisition.

The resulting peptides were loaded onto an EvoSep One (EvoSep) chromatograph coupled online to a TIMS tof Pro mass spectrometer (Bruker), that uses Parallel Accumulation Serial Fragmentation (PASEF) acquisition to provide extremely high speed and sensitivity. The 30 SPD protocol (∼44 min runs) was used, under default Evosep settings. Data-independent acquisition (DIA) was used for the acquisition of data.

DIA data were processed with DIA-NN^28^ software using default parameters. Searches were carried out against a database consisting of human protein entries from Uniprot in library-free mode. Carbamidomethylation of cysteines was considered as fixed modification and oxidation of methionines as variable modification. Data were loaded onto Perseus platform^29^ for data processing (log2 transformation, imputation) and statistical analysis (Student’s *t*-test). Proteins with a *P* < 0.05 and a fold change >2 in patients were considered for further analyses and discussion. Functional annotation and enrichment analysis of proteomic data was performed with DAVID bioinformatic resource.^30^

### *Drosophila melanogaster* model and functional assays

A *Drosophila melanogaster* strain silencing the *Snup* gene in muscle cells (*UAS-iSnup-Mhc-GAL4*) was generated through the muscle-specific *Mhc* promoter, which expresses the *Drosophila* ortholog of myosin heavy chain. As control, the strain *UAS-+-Mhc-GAL4* was used. To generate these genotypes, the stocks were acquired from Vienna Drosophila Resource Centre (VDRC) (i*Snup*, #40997) and Bloomington Stock Center (Control, #35784 and Mhc-GAL4, #55133). RNA was isolated from four pools of five thoraces for each genotype, using the miRNeasy kit (QIAGEN). *Snup* mRNA expression level was measured by RT-qPCR using SYBR™ Green PCR Master Mix (Applied Biosystems). Primers used are listed in Supplementary Table 2.

Climbing and longevity assays were performed in parallel in the same population. To perform the assays, same age female flies were housed in tubes in groups of five for the duration of the experiment. The experiment was performed with a total of *n* = 60 i*Snup* flies (12 tubes) and *n* = 70 controls (14 tubes). Because of censored subjects, the total number of flies analysed in the longevity assay was of *n* = 52 in both i*Snup* and controls. Flies were housed at 24°C, 70% humidity and a 12 h/12 h light/darkness cycle.

Flies were tested for locomotor activity every 5 days. The number of flies that crossed an 8 cm line in 10 s was counted and represented as the percentage of climbing flies per tube (*n* = 12 i*Snup*; *n* = 14 Controls). The procedure was repeated three times at each time point and the mean percentage of flies was calculated. The mean climbing rate was compared between strains through *t*-tests. For longevity assays, the status of the flies was checked three times per week.

### Bioinformatic and statistical analyses

Raw reads obtained from RNA sequencing experiments were filtered to remove adaptor sequences, contamination and low quality reads.

For quantification of gene expression, RNA sequencing reads were mapped with *Salmon* (v0.14.1).^31^ TPM counts were aggregated per gene using *rtracklayer* (v1.58.0). Differential gene expression analysis was performed with *DESeq2* (v1.38.3),^32^ where raw gene counts were normalized with VST. Genes were considered differentially expressed if the adjusted *P-*value was <0.05 and the absolute shrinked log fold change, calculated with *apeglm* method from *apeglm*(v1.20) packages,^33^ was >2. For Gene Ontology analysis *clusterProfiler2* (v.4.8.1) Bioconductor package was used, along with human annotation database *org.Hs.eg.db* (v3.17.0) available through Bioconductor.

To quantify the expression of snRNA genes, RNA sequencing reads were mapped to the GRCh38transcriptome using *STAR* (v2.7.7a),^34^ allowing for multi-mapping (*−outFilterMultimapNmax 100*). Expression was quantified with *featureCounts* (v2.0.1), allowing multi-mappers with the *-M and -F* command-line flags. Differential gene expression analysis was then carried out using *DESeq2* (v1.40.2),^32^ after normalization with the VST method. Genes with an adjusted *P*-value < 0.05 threshold and an absolute log2 fold change >2 were considered as differentially expressed. These results were then further processed by merging with annotations obtained from the *BiomaRt*(v2.56.1) Bioconductor package (snRNA biotype filter).

Alternative splicing analysis was performed using *vast-tools* (v2.5.1).^35^ FASTQ reads were aligned with *vast-tools align* using human VASTDB library (vastdb.hs2.23.06.20). Differentially regulated events were identified using *vast-tools compare*, with default parameters: |ΔPSI| > 15 between the means of the two compared groups (*—min_dPSI 15*), and a non-overlapping PSI distribution between two sample groups of at least 5 (*—min_range 5*). Differentially spliced events in differentially expressed genes were identified by filtering the list of differentially regulated events to those within differentially expressed genes. To identify differentially regulated events overlapping in both patients, first we assessed changes in inclusion per patient under the same thresholds as above and then filtered those showing a correlation of 0.9 in their |ΔPSI| between patients relative to the controls.

To identify muscle enriched splicing events, we performed tissue pairwise comparisons in publicly available VastDB database.^35^ Inclusion table for human was downloaded from VastDB (https://vastdb.crg.eu/). We complemented these data with the control muscle RNA-seq dataset from this study as additional input. To define muscle-enriched alternative splicing programme, we used the ‘*Get_Tissue_Specific_AS.pl*’ script, with the following parameters: (i) absolute difference in the average event inclusion level between the target tissue and the average across other tissues of |PSI| > 15; (ii) global |PSI| > 25—the difference between target tissue inclusion average and the average of all other tissues as one group (*—min_dPSI_glob 25*); (iii) a valid average PSI value in at least *n* = 5 tissues (—*N_groups 10*); and (iv) sufficient read coverage in at least *n* = 3 samples per valid tissue group (*—min_rep 3*)—score VLOW or higher as provided by *vast-tools*. The list of events was further filtered by computing the ΔPSI between muscle and each of the other tissues individually and selecting events with ΔPSI *>* 15 along all of the tissues versus muscle.

Alternatively spliced events in genes encoding spliceosome components were identified by filtering the list of differentially regulated events overlapping in both patients with a curated list of human splicing factors and regulators.^36^ Sashimi plots comparing alternative splicing events between patients and controls were generated with *ggsashimi.*^37^

Statistical analyses were performed using the GraphPad Prism 8.0.1 for Windows (GraphPad Software). Student’s *t*-test was used when analysing differences between two groups. More than two group comparisons were performed with one-way ANOVA followed by multiple comparisons with a control group. Dunnett’s correction for multiple comparisons was applied. The survival analysis was performed using the Kaplan–Meier method and assessing the differences between groups with the Mantel-Cox test. The alpha level for statistical significance was set at < 0.05.

## Results

### Patients with *SNUPN* variants show a LGMD phenotype with myofibrillar-like features

**Figure 1:**
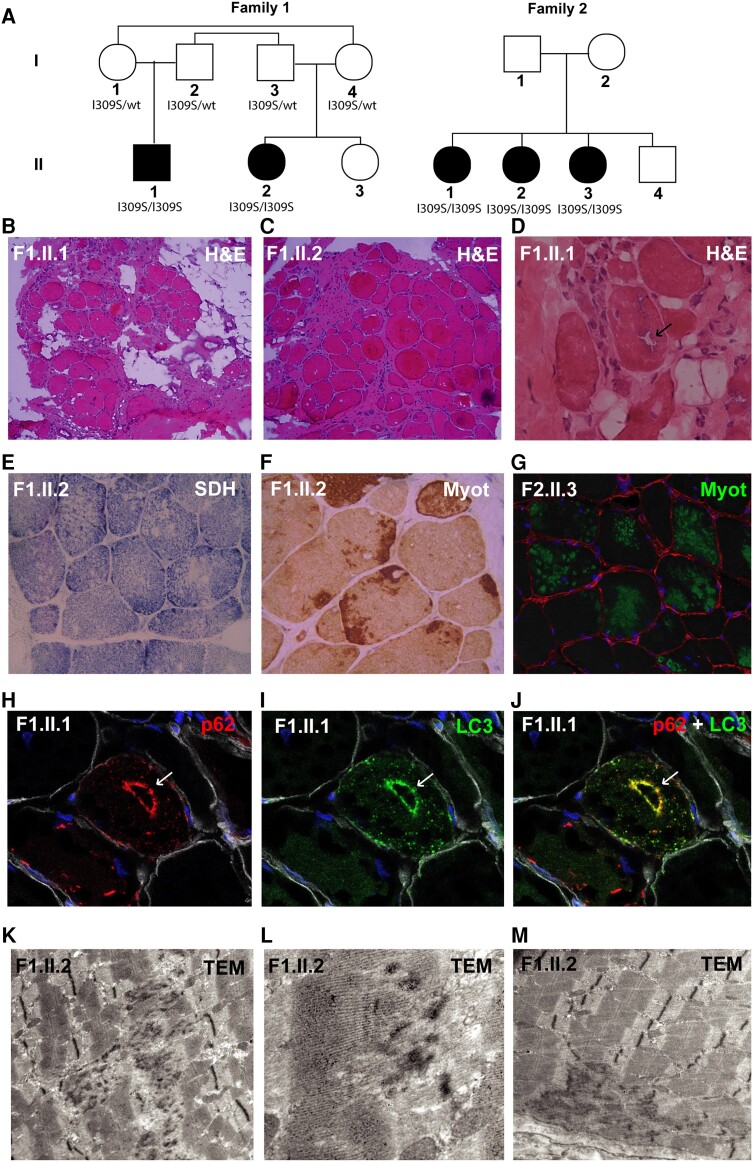
**Patients with *SNUPN* variants show a LGMD phenotype with myofibrillar-like features.** (**A**) Pedigree of the two families included in this study. (**B**–**D**) Histopathological alterations in Patients II.1 and II.2 from Family 1 by haematoxylin and eosin (H&E) staining, which mainly show marked increase in endomysial adipose and connective tissue (**B** and **C**, ×10) and rimmed vacuoles (black arrow) (**D**, ×40). (**E**) Succinate dehydrogenase histochemical (SDH) staining shows minicore-like features (×20). (**F** and **G**) Frequent myotilin sarcoplasmic aggregates are present (×20). Green = myotilin; red = laminin; blue = DAPI. (**H**–**J**) p62 and LC3 aggregates are also found, sometimes co-localizing around vacuoles (white arrows) (×40). Red = p62; green = LC3; white = laminin; blue = DAPI. (**K** and **L**) Prominent Z-line streaming and disorganization are shown by transmission electron microscopy (TEM). In **M**, focal areas of myofibril disruption and absence of mitochondria, minicore-like, are also identified. LGMD = limb girdle muscular dystrophies; wt = wild-type.

The clinical features of five patients from two unrelated families carrying a biallelic variant in *SNUPN* gene are reported in Table 1. The proband (Patient F1.II.1) from Family 1 is a 17-year-old male who started with pelvic limb girdle weakness at 4 years of age, reporting difficulty in climbing stairs and frequent falls. At 9 years old, he started with shoulder girdle impairment, difficulties walking on his toes and severe respiratory restrictive impairment. In examination, positive Gowers’ sign, predominantly proximal weakness, hyperlordosis, equinovarus feet and contractures were found. No facial involvement was identified. In the last examination, at 17 years of age, the patient was wheelchair-bound, with severe weakness and restrictive respiratory dysfunction. Regarding family history, his parents are both healthy, living in Romania and from Caucasian ancestry. He has a double cousin (Patient F1.II.2) who is also affected (Fig. 1A). Patient F1.II.2 started with pelvic girdle weakness at the age of 9. At 13 years of age, she underwent surgical correction for equinovarus feet. In the last examination (20 years of age), she had difficulties walking on her toes, hyperlordosis and moderate restrictive ventilatory dysfunction. Facial muscle weakness was not present. Initial ancillary tests showed elevated creatine kinase (CK) levels (between 1500–5750 IU/l). Nerve conduction studies were normal in Patient F1.II.1 and showing slightly reduced compound muscle action potential amplitude, likely related to muscle atrophy, in Patient F1.II.2. EMG showed a myopathic pattern in the proband.

**Table 1:** Clinical features of patients with variants in *SNUPN*

Family/patient	F1.II.1	F1.II.2	F2.II.1	F2.II.2	F2.II.3
Origin	Romania	Romania	Macedonia	Macedonia	Macedonia
Gender	M	F	F	F	F
Variant (protein)	p.Ile309Ser	p.Ile309Ser	p.Ile309Ser	p.Ile309Ser	p.Ile309Ser
Zygosity	HOM	HOM	HOM	HOM	HOM
Frequency genomAD (v4)	0.000005472	0.000005472	0.000005472	0.000005472	0.000005472
Age at onset	4 years	9 years	9 years	1 year	2 years
Age at last examination	17 years	20 years	29 years	25 years	24 years
Symptoms at onset	Difficulty climbing stairs	Difficulty running	Elbow contracture and rigid spine	Difficulties in getting up from the floor and climbing stairs	Hand weakness, difficulties climbing stairs
Course	Progressive	Progressive	Progressive	Progressive	Progressive
Neurodevelopmental delay	No	No	No	No	No
Lower limb weakness	Yes, p > d	Yes, p > d	Yes, p > d	Yes	Yes, p > d
Upper limb weakness	Yes, p > d	Yes, p	Yes	Yes	Yes
Facial muscle involvement	No	No	No	No	No
Hyperlordosis	Yes	Yes	Yes	Yes	Yes
Contractures	Yes (generalized)	Yes (feet)	Yes (generalized)	Yes (generalized)	Yes (neck)
Hyperlaxity	No	No	No	No	Yes
Respiratory involvement	Restrictive ventilatory dysfunction	Restrictive ventilatory dysfunction	Restrictive ventilatory dysfunction	Restrictive ventilatory dysfunction	Restrictive ventilatory dysfunction
Cardiac involvement	Incomplete right bundle block	No	No	No	No
Walking aid	Wheelchair	No	Walker	Wheelchair	No
CK (IU/l)	1900–5750	1500–2600	1800	2300	290–350
Muscles affected in MRI	Glutei, vasti, sartorius, gracilis, semitendinosus	Gllutei medium, vasti, sartorius, gracilis	Infraspinosus, supraspinosus, deltoids, biceps, paravertebral, sartorius, gracilis, adductor magnus, vasti, peroneal, medial gastrocnemiaus	Severely affected with extensive fat infiltration, including arms, paravertebral, thighs and legs	Infraspinosus, paravertebral, glutei medius, peroneal, tibialis anterior, medial gastrocnemius, solei
Myotilin deposits	Yes	Yes	N/A	N/A	Yes
Electron microscopy	Z-disc disorganisation,	Z-disc disorganisation,	N/A	N/A	Z-disc disorganisation
EMG	Myopathic	N/A	N/A	N/A	N/A
Nerve conduction studies	Normal	Reduced amplitude probably related to muscle atrophy	Normal	N/A	N/A
Other	–	–	Alport syndrome	Alport syndrome	Alport syndrome, myalgia

F = female; M = male; HOM = homozygous; p = proximal; d = distal.

Proband in Family 2 (Patient F2.II.1) is the oldest of four siblings from non-consanguineous parents from Macedonian origin (Fig. 1A). She started with elbow contractures and difficulty climbing stairs at the age of nine. In the last examination, at 29 years old, there were severe contractures in neck, upper and lower limbs and she needed a walker. Nerve conduction studies were normal. Her two younger sisters are also affected (Patients F2.II.2 and F2.II.3). Patient F2.II.2 had difficulties walking since the age of one. She lost ambulation at 13 years of age. In the last examination, at 25 years of age, she showed generalized contractures including neck flexion contracture, predominant lower limb weakness was present and she was wheelchair-bound. CK levels in both sisters were significantly increased (1800–2500 IU/l). The youngest sister (Patient F2.II.3) presented with hand weakness at the age of 2. She reported difficulty climbing stairs in the last examination at the age of 24, but was still able to walk independently. Her CK levels were only slightly elevated (280–350 IU/l) and she had only a neck contracture. Remarkably, all three sisters show severe respiratory insufficiency requiring nocturnal non-invasive ventilation. None of them presented with facial weakness.

#### Muscle biopsies

Muscle biopsies were analysed from both patients in Family 1 (quadriceps) and from Patient F2.II.3 (tibialis anterior) and histopathological reports were revised from Patient F2.II.2 (quadriceps). Histological analyses showed altered muscle architecture due to marked endomysial fibrous and fatty infiltration. Some necrotic fibres were detected. There was variability in fibre size and increased internalized nuclei (Fig. 1B and C and Supplementary Fig. 1A and B). Scarce rimmed vacuoles were present in muscle biopsies from Family 1 (Fig. 1D). With oxidative histochemical techniques, some fibres showed uneven oxidative enzyme staining, with minicore type features (Fig. 1E and Supplementary Fig. 1C). Whorled fibres were also identified in Family 1 (Supplementary Fig. 1A). Interestingly, predominant subsarcolemmal and central aggregates of Myotilin were identified in both families (Fig. 1F and G and Supplementary Fig. 1D). P62 (Fig. 1H and Supplementary Fig. 1E) and LC3 (Fig. 1I and Supplementary Fig. 1F-G) aggregates were also found, both in myofibrillar disintegration areas and co-localizing in rimmed vacuoles (Fig. 1J). Transmission electron microscopy was performed in both patients from Family 1 and one in Family 2 (Patient F2.II.3) and showed prominent Z-line streaming and disorganization (Fig. 1K and L). Focal areas of myofibril disruption and a scarcity or absence of mitochondria, minicore-like, were also identified (Fig. 1M). Additional histological studies are shown in Supplementary Fig. 1H and I. In conclusion, from a histopathological perspective, these findings suggest a muscular dystrophy with myofibrillar-like features.

#### Muscle MRI

**Figure 2:**
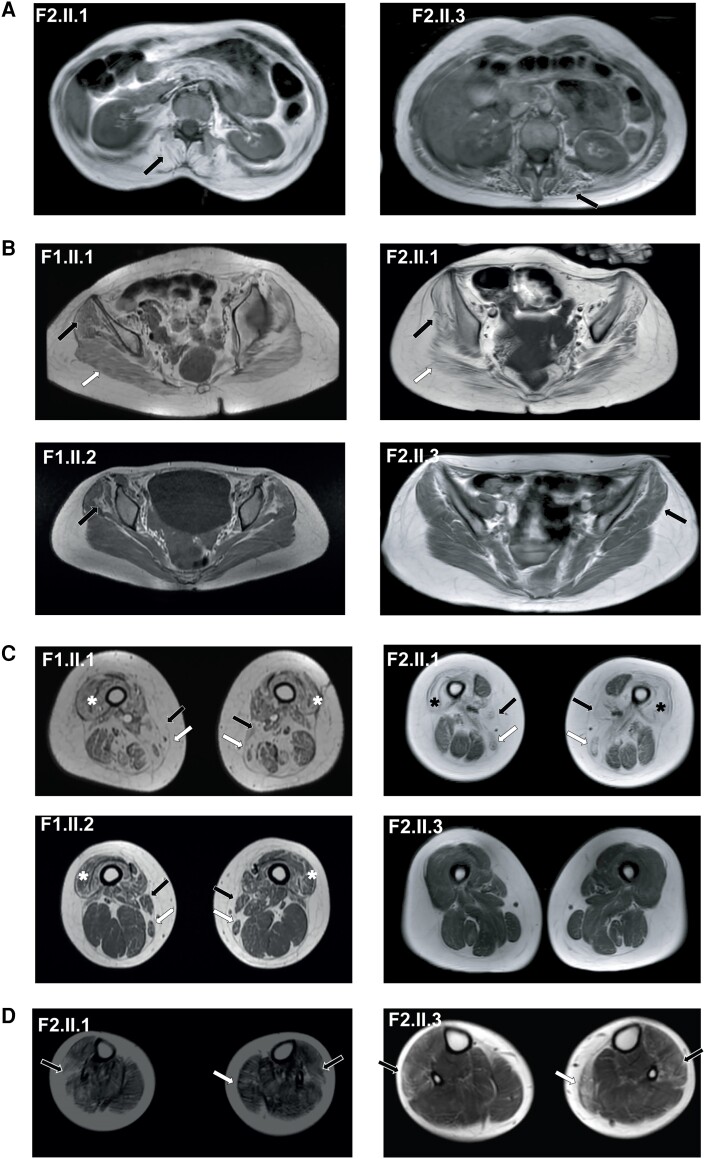
**Patients with *SNUPN* variants show a common pattern in MRI.** (**A**) Paravertebral muscles were commonly replaced by fat (black arrow). (**B**) Gluteus medius (black arrow) was also affected, involving glutei magnus in more severe phenotypes (white arrow). (**C**) Primarily involved muscles at the thighs included vasti intermedius, vasti lateralis (asterisk), vasti medialis, adductor magnus, sartorius (white arrow) and gracilis (black arrow). (**D**) In legs, affected muscles were predominantly peroneus longus (black arrow) and medial gastrocnemius (white arrow) although tibialis anterior was also involved in Patient F2.II.3.

Muscle MRI was performed in all patients, including total body MRI in patients from Family 2 and from pelvis to knees in those from Family 1. Patient F2.II.2 showed generalized severe fat replacement. Paravertebral muscles were commonly replaced by fat in patients from Family 2 (Fig. 2A). Glutei medius were involved in all the patients and glutei magnum in those more severely affected (i.e. Patients F1.II.1 and F2.II.1) (Fig. 2B). Primarily affected muscles at the thighs comprised vasti intermedius, vasti lateralis, vasti medialis, adductor magnus, sartorius and gracilis in four patients (Fig. 2C). Remarkably, posterior compartment muscles were commonly less affected or even spared, as well as rectus femoris in Patient F2.II.1. In legs (only available in Family 2), primarily affected muscles were peroneus longus and medial gastrocnemius, although tibialis anterior was also involved in Patient F2.II.3 (Fig. 2D). No changes suggestive of active inflammation were detected on short tau inversion recovery (STIR) sequences. Additional details of the muscles involved in each patient are shown in Table 1.

### Genetic and structural characterization of *SNUPN* variants

WES analysis did not identify any clinically relevant variant in genes already known to cause neuromuscular disease. However, a variant of uncertain significance was found in *SNUPN* (ENSG00000169371) in both families (Fig. 2A). All patients were homozygous for a missense variant in exon 9 (c.926T>G; NM_0011042581; p.Ile309Ser) (Supplementary Fig. 2A and B). This variant (GRCh38 Chr15:g.75598515A>C), was present at a very low frequency in gnomADv4 (0.000005472) and segregated with the disease in Family 1 (Supplementary Fig. 2A). Other recessive or copy number variants shared by both families were excluded by WES analysis in Patients F1.II.2 and F2.II.1 (Supplementary Table 4).

**Figure 3:**
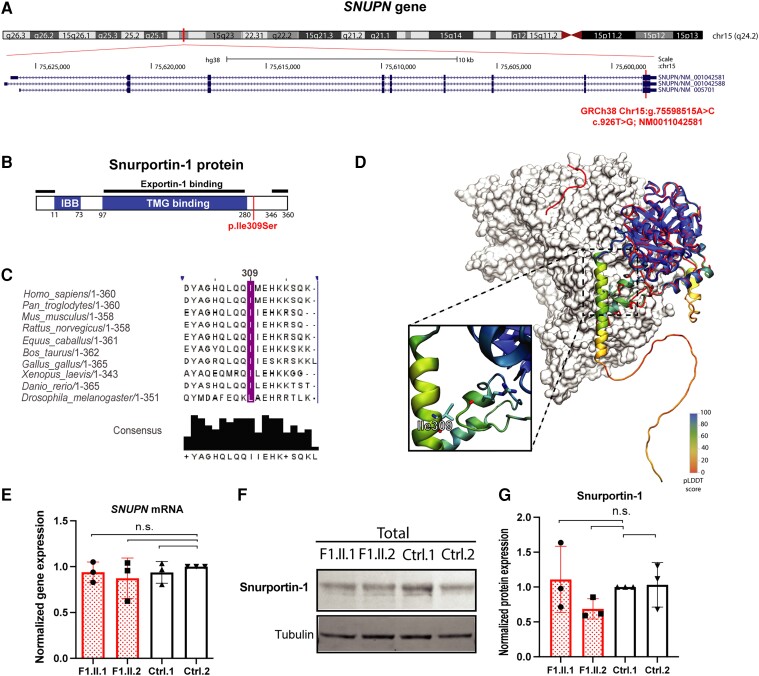
**Genomic, conservation, expression and protein analysis of *SNUPN* variant.** (**A**) UCSC genome browser view of *SNUPN* gene in Chr15. The position where the variant is located in exon 9 is highlighted in red. (**B**) Graphical structure of Snurportin-1 protein with its more important domains depicted as IBB (Importin-β binding domain), TMG binding (trimethylguanosine binding domain) and Exportin-1 binding. The amino acid that is mutated in patients is labelled in red. (**C**) Protein alignment and amino acid conservation of p.Ile309 residue and surrounding sequence. (**D**) Cartoon representation of the full-sequence Alphafold model of Snurportin-1 (AF id: O95149), coloured by pLDDT value, overlaid on the export complex (PDB id: 3gjx, Snurportin-1 in red and exportin-1 as white surface) and the m3G-cap-binding domain (PDB id: 1xk5, blue). The *inset* highlights the location of Ile309 residue with atomic detail. (**E**) Expression level of *SNUPN* mRNA by qPCR in patient (Patients F1.II.1 and F1.II.2) and control fibroblasts. Quantification was performed in RNA samples isolated from three different cellular passages and normalized against TBP expression level. (**F**) Representative western blot image of Snurportin-1 protein level in patient (Patients F1.II.1 and F1.II.2) and control fibroblasts. (**G**) Quantification of normalized Snurportin-1 protein levels from three independent western blot experiments performed using total cellular lysates isolated from three different cellular passages. The expression level is normalized against Tubulin and relative to the expression level in Ctrl-1. n.s. = not significant.

*SNUPN* gene encodes Snurportin-1 (NP_001036046), a 360-residue protein that functions as an U-snRNP nuclear import adapter (UniProtKB ID: O95149) (Fig. 3B). The variant affects a highly conserved residue in all vertebrates from human to zebrafish. In *Drosophila*, p.Ile309 amino acid changes to the highly homologous Leu (Fig. 3C). Moreover, *in silico* protein prediction analysis with Polyphen and SIFT indicated a negative impact of the variant on the protein (Supplementary Fig. 2C and D). CADD score, a widely used measure of variant deleteriousness, was 28.9 and AlphaMissense rate was 0.9, further supporting its pathogenicity. The presence of p.Ile309Ser variant did not alter *SNUPN* mRNA levels in blood in Family 1 (Supplementary Fig. 2E). Therefore, we hypothesized that the variant may affect protein structure, stability and/or function rather than leading to haploinsufficiency.

The Ile309Ser mutation is located close to the C-terminus of Snurportin-1, within a region that has not been resolved in any of the available experimental structures. The C-terminal domain of Snurportin-1 is predicted to be an intrinsically disordered region (IDR), which includes residue 309 according to IUPRED2a prediction server (Supplementary Fig. 2F). This prediction did not differ substantially for the Ile309 variant. Importantly, other servers predicted the disordered region starts a few residues after Ile309 in the Snurportin-1 sequence. On the other hand, in the Alphafold model, part of the C-terminal region is predicted with moderate confidence to fold as a long α-helix (residues 298–325; Fig. 3D) that forms contacts with the globular domain of the protein. Finally, in the Snurportin-1 complexes available with Exportin-1,^16^ a few residues at the C-terminus were modelled far from the m3G-cap-binding domain but forming interactions with Exportin-1 (Fig. 3D), suggesting that the C-terminal region of the protein where the variant is located undergoes high amplitude motions. Both the formation of intra and intermolecular interactions will be affected by the mutation due to the lower hydrophobicity of serine. In a well curated database of free energy changes upon mutation, changes from Ile to Ser resulted in an average destabilization of 13 kJ/mol (twice the mean of the dataset).^38^

### Patients show cytoplasmic accumulation of snRNP components

To investigate the functional consequences of the variants identified in *SNUPN*, we isolated fibroblasts from skin biopsies in Patients F1.II.1 and F1.II.2 and two unrelated healthy controls (Supplementary Table 1). First, we confirmed that the variant does not change *SNUPN* mRNA levels in patients (Fig. 3E). The variant did not modify the overall expression of Snurportin-1 protein either (Fig. 3F and G). We then measured whether the variant could affect the expression of snRNP proteins. Specifically, we looked into snRNP-specific proteins within U1 (U1A and U170K), U2 (U2A’ and U2B’’) as well as the core Sm protein SmB/B’ and SMN. Our results indicate that the overall expression of snRNP-specific proteins was not affected in patients (Supplementary Fig. 3A and B). However, certain proteins, such as U1A, showed a slight but non-statistically significant increase in patients (Supplementary Fig. 3B). Similarly, total expression of U-snRNAs (including U1, U2, U4, U5 and U6) remained unchanged (Supplementary Fig. 3C).

**Figure 4:**
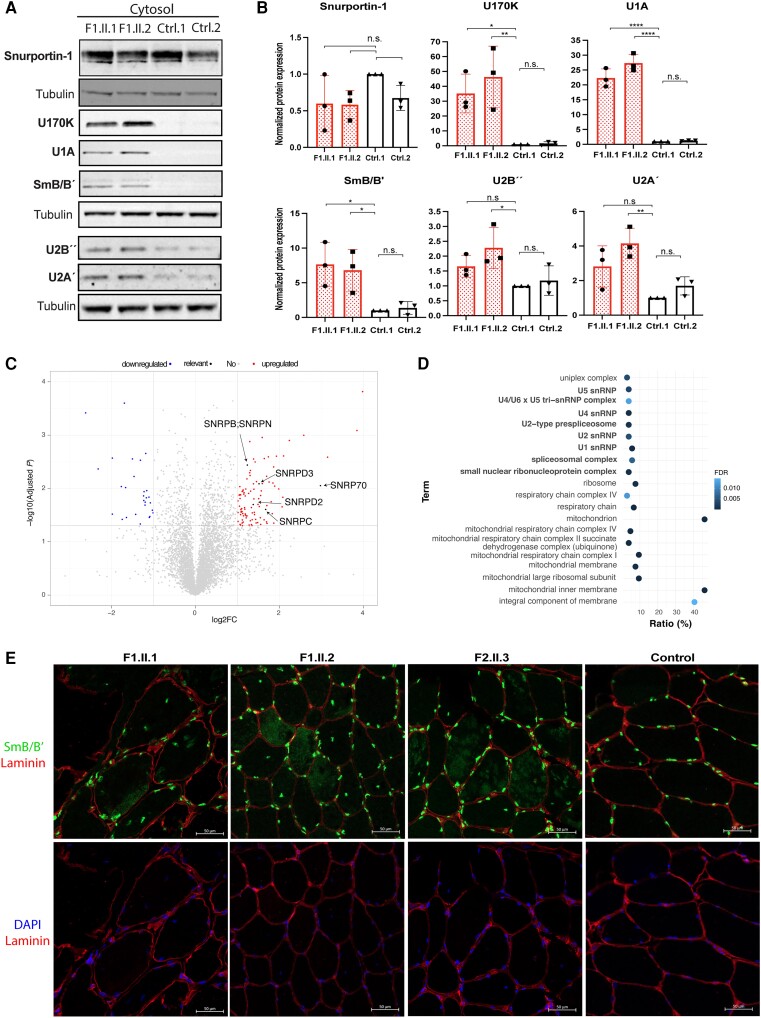
**Patients show cytoplasmic accumulation of snRNP components**. (**A**) Representative western blot images of Snurportin-1, U1-70 K, U1A, SmB/B´, U2B´´ or U2A´ U-snRNP proteins in the cytosolic fractions of patient and control fibroblasts. (**B**) Quantification of normalized protein levels shown in **A**. Values were obtained from three independent western blot experiments performed in lysates from three different cellular passages. The expression level is normalized against Tubulin and relative to the expression level in Ctrl1. n.s. = non-significant. *P < 0.05, **P < 0.01, ****P < 0.0001. (**C**) Volcano plot representing up- (red) and downregulated (blue) proteins in patient’s cytosolic fractions compared to controls. (**D** Gene Ontology analysis with upregulated proteins according to cellular component. (**E**) Representative images of SmB/B’ immunofluorescence in patients’ and control muscle biopsies. snRNP = small nuclear RNA-binding protein.

As Snurportin-1 mediates the active nuclear import of U-snRNPs by the importin-β receptor pathway,^11,12^ we checked whether rather than a significant change in the expression of snRNP specific proteins, there was a change in the subcellular localization of these proteins. Hence, we performed a nuclear-cytoplasmic fractionation of fibroblasts followed by western blot (Supplementary Fig. 3D). Relevantly, an increase in certain U-snRNP specific proteins and the Sm core protein SmB/B’ was found in the cytoplasmic fraction of patient fibroblasts (Fig. 4A and B). The increased abundance of snRNP proteins in the cytosolic fractions of patient-derived fibroblasts was also confirmed by mass spectrometry. This analysis revealed 28 downregulated proteins (fold-change <0.5, *P-*value < 0.05) while 102 were more abundant in patients compared to controls (fold-change > 2, *P-*value < 0.05) (Fig. 4C and Supplementary Table 5). A functional annotation and enrichment analysis with overexpressed proteins identified terms related to mitochondrial function, snRNP biology and spliceosomal complex (Fig. 4D). Furthermore, SmB/B’ immunofluorescence in muscle biopsies from Patients F1.II.1, F1.II.2 and F2.II.3 showed cytoplasmic aggregates not present in control muscle (Fig. 4E). Therefore, our results indicate that biallelic p.Ile309Ser variant in *SNUPN* leads to an accumulation of U-snRNP components in the cytoplasm of patient-derived fibroblasts and muscle.

### Patients show widespread splicing deregulation in muscle relevant genes and spliceosome components

**Figure 5:**
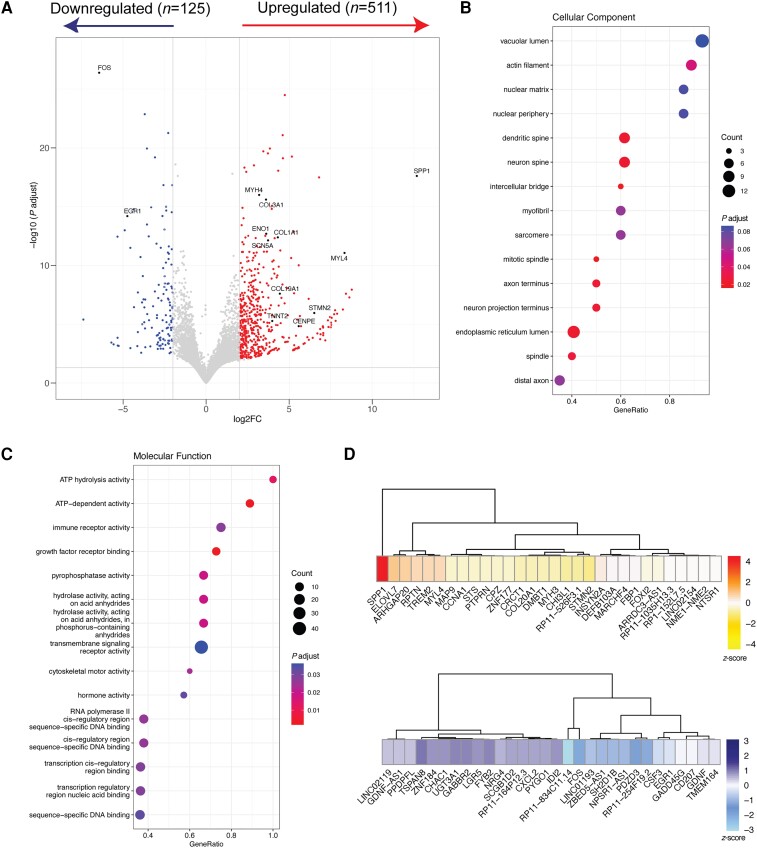
**Gene expression analysis in patients’ muscle by RNA-Seq analysis.** (**A**) Volcano plot showing up- and downregulated genes in patients compared to controls. (**B** and **C**) Gene Ontology analysis with differentially expressed genes according to cellular component (**B**) or molecular function (**C**). (**D**) Heat map depicting the top 30 overexpressed (*top*) and downregulated (*bottom*) genes in patients compared to controls.

Due to the important role of Snurportin-1 in the biogenesis of snRNPs, which are essential elements of the spliceosome complex, we analysed total RNA-seq data from muscle biopsies obtained from Patients F1.II.1 and F1.II.2, as well as three non-pathological muscles as controls (Supplementary Table 1). Gene expression data from control and patient samples clustered together in a principal components analysis (PCA) plot analysis (Supplementary Fig. 4A). First, we looked at the changes in gene expression data, where we identified 511 upregulated and 125 downregulated genes (absolute log2 fold change >2 and corrected *P-*value < 0.05) (Fig. 5A and Supplementary Table 6). Conversely, variants in *SNUPN* did not impair the overall abundance of U-snRNAs (Supplementary Fig. 4B), as previously observed in patients’ fibroblasts (Supplementary Fig. 3C). To dissect how the differentially expressed genes affect muscle function in patients’ tissue, we performed Gene Ontology (GO) analysis with differentially expressed genes (DEGs) (Fig. 5B and C). In cellular component annotation, myofibril and sarcomere appeared as two of the represented terms, but GO terms related to neuron biology (i.e. dendritic and neuron spine, neuron projection and axon terminus or distal axon) were also enriched. Indeed, expression of *STMN2*, a regulator of microtubule stability exclusively expressed in motor neurons, was restricted to patients (Supplementary Fig. 4C). Similarly, molecular function annotation revealed an impairment of many metabolic pathways, including ATP-dependent activity, hydrolases or cytoskeleton-related processes (Fig. 5C). Furthermore, several collagen-related genes were also significantly overexpressed in patients. The top 30 DEGs in each group were further extracted and plotted to assess their importance in muscle function and relevance in disease (Fig. 5D). Interestingly, several genes previously related to other muscular dystrophies^39-41^ were amongst the top overexpressed (i.e. *SPP1*) and downregulated genes (i.e. *FOS* and *EGR1*).

**Figure 6:**
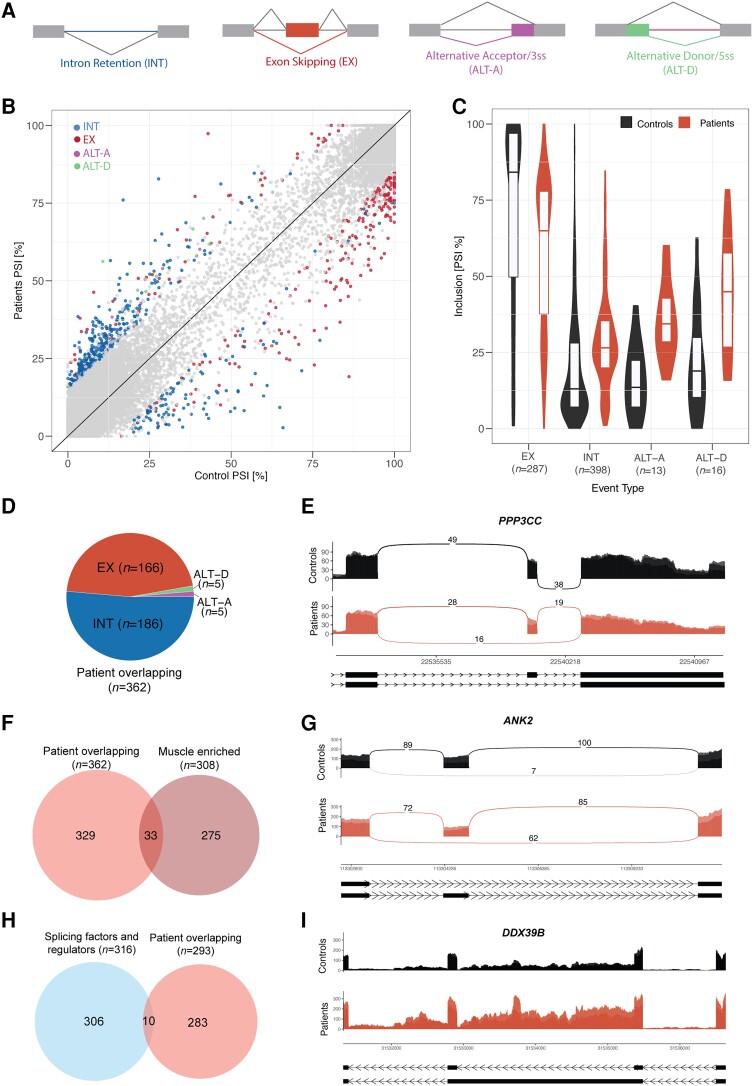
**Patients show widespread splicing deregulation in muscle relevant genes and spliceosome components.** (**A**) Diagram depicting different types of splicing events. EX = exons; INT = introns; ALT-A = alternative acceptor; ALT-D = alternative donor. (**B**) Scatter plot representing inclusion of differentially spliced events in controls (*x*-axis) and patients (*y*-axis) colour-coded by type of event. (**C**) Inclusion distributions in controls and patients per event type of differentially regulated events from **B**. (**D**) Pie chart showing the distribution of differentially regulated events by type of event overlapping in Patients F1.II.1 and F1.II.2. (**E**) Sashimi plot representing the differentially regulated event in *PPP3CC* gene. (**F**) Venn diagram showing the overlap between muscle-enriched and patient-overlapping differentially regulated events. (**G**) Sashimi plot representing the differentially regulated event in *ANK2* gene. (**H**) Venn diagram showing the overlap between genes containing patient-overlapping differentially regulated events and genes encoding splicing factors and regulators. (**I**) Sashimi plot representing the differentially regulated event in *DDX39B* gene.

We then investigated changes in alternative splicing (AS) (Fig. 6A), where again patient and control samples clustered together (Supplementary Fig. 5A). Using a threshold of minimum change in inclusion (ΔPSI) of ± 15, we identified 714 differentially spliced events within 555 genes in patient versus control group, with the majority represented by regulated introns and exons (Fig. 6B and Supplementary Table 7). Interestingly, patients showed mostly intron retention (IR) and exon skipping (EX) (Fig. 6C). However, only 11 out of the 555 genes that undergo alternative splicing had an impact in gene expression, suggesting independent mechanisms of transcriptional and post-transcriptional regulation (Supplementary Fig. 5B and Supplementary Table 8).

We then filtered for those alternative splicing events, which overlapped between patients, where we identified 362 overlapping events (Fig. 6D, Supplementary Fig. 5C and Supplementary Table 9). Patients’ muscle showed deregulated splicing transitions relevant for human skeletal muscle development and/or previously involved in other muscular dystrophies. For instance, skipping of exon 13 in *PPP3CC* gene was observed in patients, which would lead to the expression of a foetal calcineurin A isoform, which slows the timing of muscle relaxation (Fig. 6E).^42^ In *ATP2A1* gene, patients expressed a SERCA1b neonatal isoform, also misregulated in myotonic dystrophy (DM1) (Supplementary Fig. 6A).^43-45^ Other alternative splicing events included exon 29 skipping in *CACNA1S* gene (Supplementary Fig. 6B), previously reported to correlate with the severity of weakness in DM1 patients^46,47^ and a tissue-specific splicing event in *MEF2D* transcription factor (Supplementary Fig. 6C), previously involved in the commitment of differentiating myoblasts to activate the late-muscle gene expression programme.^48^ In the *MYBPC1* gene, two events in the NH2 and COOH termini of Myosin binding protein C, previously described in distal arthrogryposis myopathy, were also dysregulated in patients (Supplementary Fig. 6D and E).^49,50^

To further understand the role of deregulated splicing in muscle further, we identified a muscle-enriched alternative splicing programme containing 308 events (Supplementary Fig. 5D and Supplementary Table 10) and queried whether splicing events deregulated in both patients were within this programme. This analysis revealed 33 muscle-enriched alternatively spliced events (Fig. 6F, Supplementary Fig. 6F and Supplementary Table 11). In *ANK2* gene, skipping of a poorly annotated exon, which could impact the role of ankyrin-B in muscle, was remarkable in patients (Fig. 6G).^51^ Other important genes in muscle contraction-relaxation system (such as *NFATC3*, Supplementary Fig. 6F) and structure (such as *PDLIM3*, Supplementary Fig. 6G) showed specific splicing events in patients’ muscle.

Finally, we investigated whether deregulated events were affecting genes expressing spliceosome components. An overlap between the genes containing the 362 events deregulated in both patients (*n* = 293) with a list of curated human spliceosome components^36^ (*n* = 316) revealed 21 events within 10 genes (Fig. 6H and Supplementary Table 12), which mostly involved intron retention. Of note, many of them are splicing factors involved in U-snRNP assembly or participate in the early steps of spliceosome assembly (i.e. *DDX39B*, *SF3A3*, *U2AF2*, *DDX5*) (Fig. 6I and Supplementary Fig. 6H–J).

In conclusion, the splicing alterations observed in patients’ muscles suggest that variants in the *SNUPN* gene may lead to splicing deregulation in skeletal muscle.

### Reduced expression of *Snup* in *Drosophila melanogaster* impairs mobility and survival

**Figure 7:**
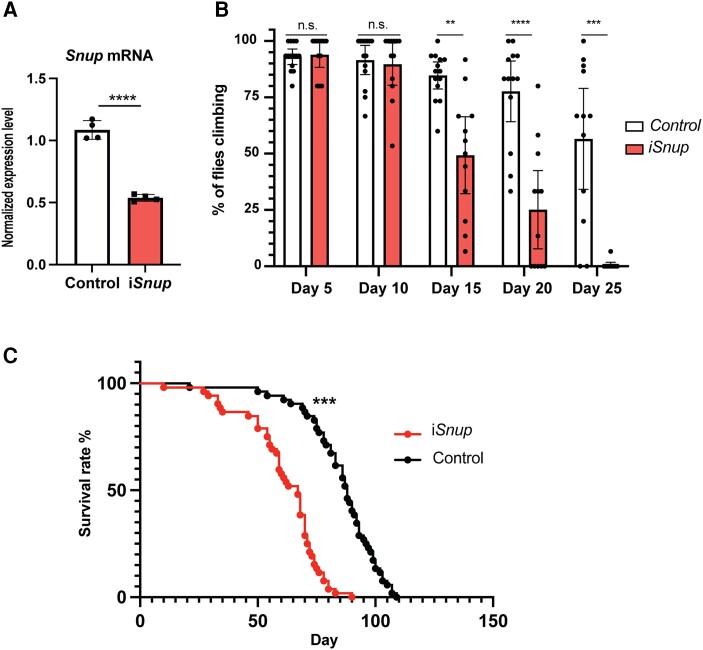
**Reduced expression of *snup* in *Drosophila* impairs mobility and survival.** (**A**) mRNA expression levels of Snup by qPCR measured in pools of thoraces in control and i*Snup* flies. (**B**) Mean percentage of climbing flies measured in control and i*Snup* flies from Day 5 to Day 25. (**C**) Longevity assay plot showing the survival rate of control and i*Snup* flies over time. n.s. = non-significant. **P < 0.01, ***P < 0.001, ****P < 0.0001.

To investigate the relevance of Snurportin-1 in muscle development and function further, we generated a muscle-specific knockdown model of *Snup*, the orthologue of human *SNUPN* in *Drosophila melanogaster*. *Snup* knockdown was conditioned to the expression of muscle-specific *Mhc* and, therefore, induces the expression of an interference RNA (RNAi) against *Snup* in a muscle-specific manner. Reduced expression of *Snup* mRNA in thorax of Day 1 i*Snup* flies was confirmed by qPCR (*P*-value < 0.001) (Fig. 7A).

Locomotor capacity was compared between i*Snup* and control flies through a climbing assay, where *Drosophila’s* negative geotaxis natural behaviour is used as a measure of proper muscle function. Both i*Snup* and control flies showed progressive age-dependent reduction in climbing activity; however, this reduction was significantly faster in the i*Snup* flies. In particular, by Day 15, the rate of i*Snup* flies passing the climbing assay [49.31%, standard deviation (SD) = 26.90] was significantly lower than the control flies (84.70%, SD = 10.39; *P-*value < 0.001), and by Day 25 locomotor capacity was almost completely impaired in the knockdown flies (0.55%, SD = 1.905) whereas ∼50% of controls were still able to climb (56.59%, SD = 35.24; *P-*value < 0.001) (Fig. 7B and Supplementary Videos 1 and 2).

Longevity studies showed that i*Snup* flies have a decreased lifespan when compared with controls, as indicated in Kaplan-Meier curves by the log-rank test (*P-*value < 0.001) (Fig. 7C). Median survival of the i*Snup* flies was 67 days [95% confidence interval (CI) (59, 70)], while it was 88 days in control flies [95% CI (83, 92)].

## Discussion

Here we report five affected individuals from two unrelated families who carry a biallelic missense variant in *SNUPN* and display a muscular dystrophy phenotype. Patients showed clinical similarities, mainly based on the proximal predominant weakness pattern and the presence of contractures. Remarkable respiratory impairment was also present in all patients whereas cardiac involvement was absent. CK ranged from 290 to 5700 IU/l. Histology showed fibrosis and fat replacement in muscle biopsy. Interestingly, we found myotilin, p62 and LC3 deposits, along with disorganized Z-disc in transmission electron microscopy. Furthermore, MRI showed main involvement of paravertebral, glutei medius, vasti, sartorius, gracilis, peroneal and medial gastrocnemius muscles. These findings suggest that *SNUPN* variants produce a new type of autosomal recessive LGMD, in line with current definition,^2^ with myofibrillar-like features. According to the Online Mendelian Inheritance in Man (OMIM) classification, it would represent LGMD R29.

*SNUPN* p.Ile309Ser variant does not affect Snurportin-1 protein levels, as shown in patients’ fibroblasts. Instead, our results suggest a functional impairment of Snurportin-1, which affects U-snRNP translocation and, hence, subcellular localization of U-snRNP components in patients. We observed accumulation of snRNP-specific proteins in the cytoplasm of patients’ fibroblasts, as well as SmB/B’ cytoplasmic aggregates in muscle biopsies from both families. Likewise, U2-snRNP specific U2A´ and U2B´´ proteins were also slightly upregulated in patients’ cytosols. Previous studies have shown that U-snRNP specific proteins can be imported to the nucleus independently of U-snRNP particles.^52-54^ However, growing evidence shows that U-snRNP specific proteins may also interact with U-snRNAs in the cytoplasm and that alterations in the structure or nuclear import of U-snRNAs can affect the cellular distribution of U-snRNP components. Hence, U170K bridges U1-snRNA to the SMN complex during the Sm core assembly process, interacting with U1-snRNP before it is imported to the nucleus.^55^ Furthermore, U1A cytosolic accumulation has been observed when U1-snRNA is truncated and unable to enter the nucleus.^56^

The precise molecular mechanism by which p.Ile309Ser variant disrupts Snurportin-1 function remains elusive; nevertheless, the data presented in this study provide a basis for generating hypotheses. Structural studies showed that the variant is conserved across species and located at the start of an IDR in the C-terminal region of Snurportin-1, whose deletion results in a lower affinity for Exportin-1.^15^ Specifically, the IDR may act as a swinging arm^57^ that permits high amplitude motions required for binding or as a flanking region^58^ to the folded domains. One hypothesis is that the drastic change in hydrophobicity introduced by p.Ile309Ser variant changes the tertiary structure of Snurportin-1 and prevents the efficient formation of export complexes, amongst others. Another interesting hypothesis is that the long helix predicted by Alphafold with moderate confidence in the C-terminal domain of Snurportin-1 is a conditionally folding region, that may acquire secondary structure only upon binding to a partner.^59^ The interactions between this conditionally folded helix with its partners would also be affected by the more hydrophilic Ser309 variant. In any case, additional studies are needed to fully understand the mechanism by which the variant alters Snurportin-1 function leading to severe disease.

Our RNA sequencing data suggest that the presence of *SNUPN* variants cause a general impairment in the splicing process. Deregulated events affect a foetal to adult isoform transition programme essential for skeletal muscle remodelling.^42^ Moreover, splicing changes were described in muscle-enriched events relevant for human skeletal muscle development and in genes encoding proteins relevant for muscle function,^42-51,60^ many of them previously described in other muscular dystrophies also known as spliceopathies, such as DM1.^43,45,47,60-63^ Finally, splicing alterations were described in genes encoding curated human spliceosome components. Most of them represent intron retention events, which may contribute to the plasticity of the transcriptome and regulate gene expression programmes by intricate regulatory mechanisms.^64-67^ These include *U2AF2*, *SF3A3* and *DDX39B*, all splicing auxiliary factors important for U2 snRNP assembly, interaction with the branchpoint and pre-mRNA splicing.^68-71^ U2-snRNP is an early component of the splicing reaction, which undergoes numerous conformational and compositional changes through its life cycle. It is therefore plausible that the intron retention events observed in *U2AF2* and *SF3A3* may have an impact on U2-snRNP function in patient muscle. This would be consistent with previous research on cross-regulatory splicing networks and the complexity of RBPs with the splicing events they co-regulate.^72,73^ Besides, *DDX5*, which also shows intron retention in patients, has been described as a co-regulator of muscle differentiation.^74^ To our knowledge, none of these alternative splicing events or RBPs have been associated with developmental or pathological processes in muscle before.

The splicing changes observed in patients carrying *SNUPN* variants could be attributed to alterations in nuclear-cytosol snRNP trafficking or downstream splicing effects associated with the perturbations of core splicing components. However, the transcriptomic changes could also be a consequence of the general muscle degradation process and further experiments will be necessary to precisely discern between disease-causing events or downstream effects in the damaged muscle. Similarly, further research will be needed to link Snurportin-1 dysfunction with the myofibrillar-like deposits and autophagic activation found in muscle biopsy. Several mechanisms might be involved, including abnormal splicing of muscle-related structural genes or pro-aggregation effect of cytosolic accumulation of snRNPs.

Other proteins involved in nuclear trafficking of spliceosome components have already been associated with muscular dystrophies. For instance, *TNPO3* heterozygous variants have been shown to cause LGMD D2 (previously known as LGMD 1F).^75-77^*TNPO3* encodes transportin-3, a member of the importin-B family, which mediates the transport into the nucleus of Ser/Arg-rich proteins, including splicing factors, such as SRSF1 or SRSF2.^4^ Regarding additional disorders that may be included in *SNUPN* differential diagnosis, paravertebral involvement and contractures are also frequent in Emery-Dreyfuss myopathies (although cardiac involvement is not present in *SNUPN* patients) or *COL6*, even though tigroid and ‘sandwich’ sign in MRI is only seen in *COL6.*^78^ As gracilis and sartorius muscles are not usually replaced in early stages of muscular diseases, they could be a clue for radiological differential diagnosis. Indeed, those muscles are commonly involved in some myofibrillar myopathies produced by mutations in *DES*, *CRYAB*^79^ and other congenital myopathies like *RYR1* or *SEPN1.*^78^

In summary, this study demonstrates that *SNUPN* variants are related to a new type of muscular dystrophy with variable phenotypes characterized by contractures, proximal weakness, respiratory involvement, p62 and myotilin aggregates and Z-disc disorganization in histopathology. As more families are described in the future, we will better understand the clinical phenotype as well as the underlying molecular mechanisms of *SNUPN*-related LGMD. Thus, *SNUPN* gene should be included in the genetic testing of patients with myopathy.

## Supplementary Material

awae046_Supplementary_Data

## Data Availability

Raw and processed data from RNA-sequencing experiments are available from Gene Expression Omnibus (GEO) (https://www.ncbi.nlm.nih.gov/geo/) with GEO accession number GSE253519.
